# Pattern of fall and its determinants among elderly patients attending the general outpatient clinic of a tertiary hospital in northern Nigeria: a cross-sectional study

**DOI:** 10.11604/pamj.2024.48.82.38639

**Published:** 2024-07-01

**Authors:** Muazu Shuaibu Ishaq, Zainab Abdulazeez Umar, Bukar Alhaji Grema, Godpower Chinedu Michael, Abdulgafar Lekan Olawumi, Mohammed Abubakar Abiso

**Affiliations:** 1 Department of Family Medicine, Specialist Hospital Gombe, Gombe State University, Gombe State, Nigeria,; 2 Department of Family Medicine, Aminu Kano Teaching Hospital, Kano, Nigeria,; 3 Department of Family Medicine, University of Maiduguri Teaching Hospital, Borno State, Nigeria

**Keywords:** Elderly, falls, fall injury

## Abstract

**Introduction:**

falls in the elderly are a neglected health problem in many societies, particularly in the developing world. Many health and social service providers are unprepared to prevent and manage falls and related injuries as they lack sufficient knowledge to identify their predisposing factors. For this reason, this study aims to identify the pattern of falls and its determinants among the elderly in northern Nigeria.

**Methods:**

a cross-sectional study was conducted among 300 elderly patients, selected by systematic random sampling. An interviewer-administered questionnaire was used. Data was analyzed using SPSS version 20. Variables were summarised using percentages and measures of central tendency/dispersion. The chi-square test was used in assessing the significance of associations between categorical variables. A p-value of <0.05 was considered statistically significant. Binary logistic regression analysis was conducted to identify determinants of falls.

**Results:**

the prevalence of falls and fall injuries was 41.4% and 25.4% respectively. The commonest pattern of fall injuries was swellings and pain (31.1%). Tripping was the commonest 60 (49.2%) cause of fall. Age (p<0.026, AOR=4.424, CI=1.192-16.424), presence of dizziness (p<0.015, AOR=0.334, CI=0.138-0.810), use of shoes with uneven (P<0.021, AOR=0.337, CI=0.133-0.851)/slippery soles (p<0.038, AOR=0.392 CI=0.162-0.948), having slippery mats (P<0.001, AOR=0.086, CI=0.039-0.192), wires/cords exposed (p=0.005, AOR=0.306 CI=0.132-0.705) on the pathways were the determinants.

**Conclusion:**

the high prevalence of falls and fall injuries signifies its importance in health care. This implies that physicians should be proactive in asking, assessing, and assisting the elderly to provide targeted interventions to potentially prevent falls.

## Introduction

The population of elderly persons is on the increase worldwide. Nigeria has the highest population of older persons in Africa and ninth in the world. The elderly are faced with many health challenges. WHO report shows that falls are the second leading cause of accidental or unintentional injury and deaths worldwide and, each year an estimated 684 000 individuals die from falls globally of which over 80% are in low- and middle-income countries [1].

Fall has been defined as unintentionally coming to rest on the ground with or without loss of consciousness and other than a consequence of sudden paralysis, epileptic seizure, or overwhelming external force [1]. Fall injury is an outcome of a fall that results in treatment or restriction in regular activities for at least 24 hours [2]. Fall is the leading cause of injury-related death among elderly persons and is one of the greatest health challenges in the care of this fast-growing population [2]. This is becoming a major issue for health and caregivers worldwide because consequences associated with it range from increased morbidity to reduction in length and quality of life and mortality. The prevalence of falls in the elderly varies from one environment to the other. This ranges from 28.7% in the United States [2], 28.4% in the United Kingdom [3], 26.9% in South Africa [4], and 24.2% in the central part of Nigeria [5]. The differences in the prevalence could be due to differences in socio-demographics. These differences necessitate a study of our environment.

The best predictor of falling is a previous fall. However, falls in elderly people rarely have a single cause or risk factor. A fall is usually caused by a complex interaction among the intrinsic (age-related decline in function, disorders, and adverse drug effects) and extrinsic factors (environmental hazards and situational factors) [6,7]. Studies have shown that the influence of these risk factors may vary from one environment to another and from one individual to the other within the same environment [7], but very few of these studies were conducted in our environment.

Falls are better prevented than treated owing to their consequences. The cornerstone of effective fall prevention is identifying the modifiable risk factors through proper evaluation and intervening with effective strategies. This study evaluated the elderly patients attending the general outpatient clinic for the risk factors and pattern of fall injuries peculiar to our environment and recommended targeted interventions based on its findings. This may contribute to the database for assessing falls in the elderly at the local level and may provide information that can be used to develop practice guidelines for the prevention of falls in our environment.

## Methods

**Study design:** it was a cross-sectional descriptive study.

**Study setting:** the study was carried out at the General Outpatient Clinic of Aminu Kano Teaching Hospital (AKTH), Kano. Aminu Kano Teaching Hospital is a tertiary institution with a 600-bed capacity. It serves Kano and neighboring states. The General Out-Patient Clinic (GOPC) is a busy primary care unit within the institution (AKTH) run by family physicians, who see and manage patients at first contact. An average of 250 undifferentiated adult patients are seen daily with the elderly constituting 25 (10%).

**Study population:** comprised of male and female patients aged 60 years and above who presented to the GOPC of AKTH during the period of the study from February to April 2016.

**Study materials:** a semi-structured interviewer-administered questionnaire which was adapted from a previous study by Adebiyi *et al*. was used [8]. The questionnaire was constructed in English, translated into Hausa, and back-translated. It was pretested using 30 (10% of the sample size) elderly patients attending the GOPC of a state specialist hospital in Kano and adequate revisions were made to ensure clarity. The questionnaire had seven (7) sections; A) socio-demographic data, B) history of fall, C) history of relevant medical conditions, D) relevant drug history, E) relevant home environment, F) physical examination findings and G) investigation results such as packed cell volume and random blood glucose were done. Random blood glucose was used to screen for diabetes mellitus instead of fasting blood glucose because up to 40% (12) of the respondents did not come back to do fasting blood glucose during the pre-test.

**Study protocol:** the sample size was calculated using the Fisher formula. A minimum sample size of 272 was obtained, but additional 10% (27) was added to accommodate for non-response, missing data, which was approximated to a total sample size of 300. A systematic random sampling method was used to recruit the 300 participants who met the inclusion criteria from the triaging hall. Participants were included if they consented to the study. Disoriented persons who could not respond to the questionnaire independently were excluded from the study because some of the questions required recall of past events or those that needed emergency care.

Participation was voluntary, and written informed consent was obtained. History of falls (self-reported) was asked using the definition of the WHO Global Report on fall prevention in older age [1]. Insomnia was assessed using the first three questions in the World Mental Health Survey version of the WHO Composite International Diagnostic Interview (CIDI) [9]. Self-reported history of relevant medical conditions in the previous 12 months were asked including hypertension, diabetes mellitus, stroke, chronic joint pain, etc. Self-reported history of medications used was also asked. Questions were also asked to assess the home environment for relevant risk factors for fall injuries. Clinical examination was done by the researcher to assess the participants for clinical risk factors of fall and fall-related injuries, these included BMI, visual acuity, timed up and go test etc.

Data was stored in a pass-worded computer and analyzed using SPSS version 20 statistical software. Absolute numbers and simple percentages were used to describe categorical variable, whereas quantitative variables were described using measures of central tendency and dispersion. Relationships were assessed using bivariate analysis. The Chi-square test and Fisher´s exact test were used in assessing the significance of associations between categorical variables. A p-value of <0.05 was considered statistically significant. Logistic regression was done to find the predictors of falls and to remove the effect of cofounders. Adjusted Odds Ratios (AOR), 95% Confidence Intervals (CI), and p-values were calculated. A p-value of <0.05 was considered statistically significant.

**Ethical consideration:** ethical approval was obtained from the medical research ethics committee of AKTH, Kano with a reference number NHREC/21/08/2008/AKTH/EC/1190 and AKTH/MAC/SUB/12A/P-3/VI/1290. The provision of the Helsinki Declaration was respected. All investigations carried-out for the purpose of this research were borne by the researcher. Participants also benefitted from health education on fall prevention and those identified with risk factors were managed or referred to other specialty clinics appropriately.

## Results

**Figure 1:**
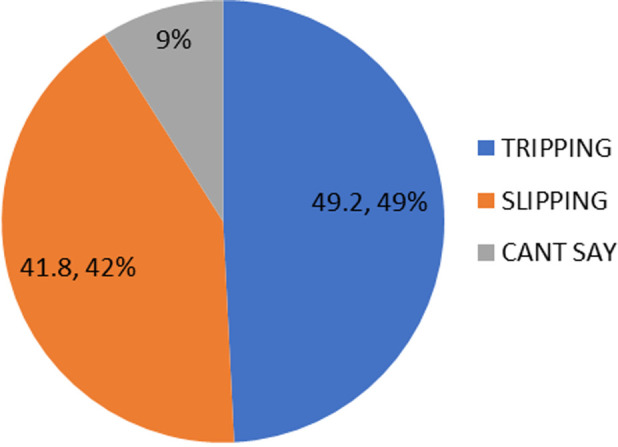
a pie chart showing the pattern of falls among those who fell

The study had a response rate of 98% (295 participants) as 5 participants had missing data from the questionnaire. The majority 194 (65.8%) of the respondents were within the age group 60-69 years, with a mean age of 67.2 + 6.4 years. Most of the respondents 162 (54.9%) were females and the majority 218 (73.9%) lived in urban areas. A significant number were married 175 (59.3%) and most of them 163 (55.3%) were from monogamous marriages. Majority of the participants 137 (46.4%) earned less than N5000 per month. Other sociodemographic characteristics are shown in Table 1. The prevalence of falls in the preceding 12 months among the study participants was 41.4%. The pattern of fall was mainly tripping in 60 (49.2%), this was followed by slipping in 51 (41.8%) of the fallers as seen in Figure 1.

**Table 1:** socio-demographic characteristics of the study participants

Characteristic	Frequency (N=295)	Percentage (%)
**Sex**		
Male	133	45.1
Female	162	54.9
**Age mean (67.2+6.4)**		
60-69	194	65.8
70-79	81	27.5
>80	20	6.7
**Residence**		
Urban	218	73.9
Rural	77	26.1
**Marital status**		
Single	6	2
Married	175	59.3
Divorce	7	2.4
Widowed	107	36.3
**Marital setting**		
Monogamous	163	55.3
Polygamous	132	44.7
**Religion**		
Islam	282	95.6
Christianity	12	4.1
Traditional	1	0.3
**Tribes**		
Hausa	234	79.3
Fulani	24	8.2
Yoruba	11	3.7
Igbo	8	2.7
Others	18	6.1
**Education**		
None	5	1.7
Quranic	207	70.2
Primary	42	14.2
Secondary	20	6.8
Tertiary	21	7.1
**Occupation**		
Civil servant	7	2.4
Trader	55	18.6
Self-employed	83	28.1
Artisan	10	3.4
Housewife	80	27.1
Retired	60	20.3
**Average income per month**		
< N 5000	137	46.4
5000-20000	117	39.7
21,000-50,000	31	10.5
51,000-100,000	10	3.4

**Figure 2:**
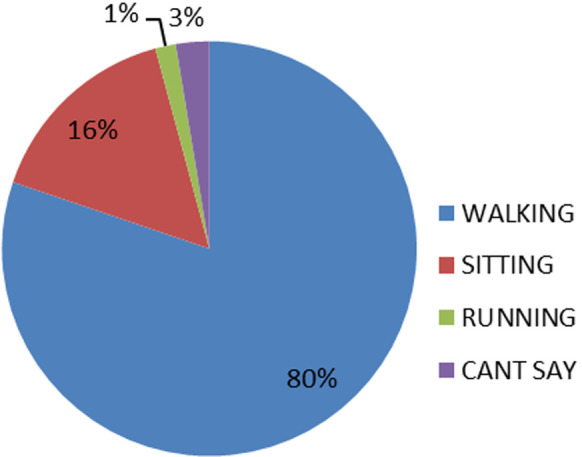
a pie chart showing activity engaged in at the time of fall

**Figure 3:**
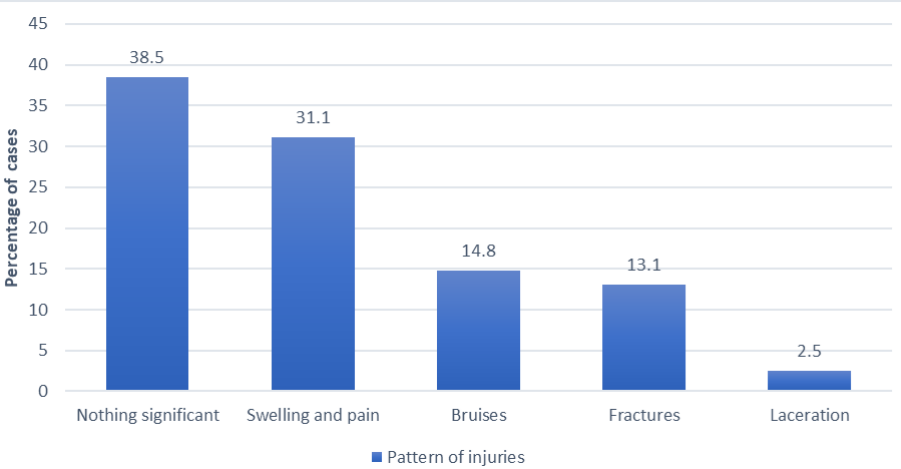
a bar chart showing the pattern of injuries among those who fell

Figure 2 shows the activity engaged at the time of falls, most of the fallers in study 98 (80.2%) experienced falls while walking. Only 3 (2.6%) could not describe the activity they were engaged at the time of the fall. Among the respondents who had falls, 25.4% had fall injuries. The pattern of injuries reported were mainly swellings and pain 38 (31.1%), while fractures were 16 (13.1%), as seen in Figure 3. At the bivariate level as seen in Table 2 and Table 2.1, age (p<0.001), sex (p<0.002) marital status (p<0.013), educational level (p<0.009), average income per month (p<0.001) were significantly associated with falls. Other medical conditions associated with falls include diabetes mellitus (p-value <0.027), chronic joint pain (p-value <0.001), and dizziness (p-value <0.007).

**Table 2:** bivariate analysis of falls and sociodemographic/medical history factors

Characteristics	Fallers n=122(%)	Nonfallers n=173(%)	Total N=295(%)	χ^2^	P-value
**Age**				16.292	<0.001*
60-69	65(53.3)	129(74.6)	194(65.7)		
70-79	43(35.2)	38(22.0)	81(27.5)		
>80	14(11.5)	6(3.5)	20(6.8)		
**Sex**				9.456	0.002*
Male	42(34.4)	91(52.6)	133(45.1)		
Female	80(65.6)	82(47.4)	162(54.9)		
**Marital status**				6.232	0.013*
Married	62(50.8)	113(65.3)	175(59.3)		
Unmarried	60(49.2)	60(34.7)	120(40.7)		
**Educational status**				12.997**	0.009*
None	1(8.0)	4(2.3)	5(1.7)		
Quranic	99(81.1)	108(62.4)	207(70.2)		
Primary	11(9.0)	31(17.9)	42(14.2)		
Secondary	7(5.7)	13(7.5)	20(6.8)		
Tertiary	4(3.3)	17(9.8)	21(7.1)		
**Income per month**				26.522**	<0.001*
<5000	73(59.8)	62(35.8)	135(45.8)		
5000-20000	43(35.2)	74(42.8)	117(39.7)		
21000-50000	3(2.5)	30(17.3)	33(11.2)		
51000-100000	3(2.5)	7(4.1)	10(3.3)		
**Hypertension**				0.001	0.997
Yes	98(80.3)	139(80.3)	237(80.3)		
No	24(19.7)	34(19.7)	58(19.7)		
**Diabetes mellitus**				4.891	0.027*
Yes	36(29.5)	32(18.5)	68(23.1)		
No	86(70.5)	141(81.5)	227(76.9)		
**Stroke**				0.273	0.601
Yes	6(4.9)	11(6.4)	17(5.8)		
No	116(95.1)	162(93.6)	278(94.2)		
**Chronic joint pain**				11.153	0.001*
Yes	89(73.0)	93(53.8)	182(61.7)		
No	33(27.0)	80(46.2)	113(38.3)		
**Insomnia**				1.421	0.233
Yes	44(36.1)	51(29.5)	95(32.2)		
No	78(63.9)	122(70.5)	200(67.8)		
**Dizziness**				7.280	0.007*
Yes	34(27.9)	26(15.0)	60(20.3)		
No	88(72.1)	147(85.0)	235(79.7)		
**Multimorbidity**				0.032	0.858
Two- Four	32(26.2)	47(27.2)	79(26.8)		
More than four	90(73.8)	126(72.8)	216(73.2)		
**Antihypertensive**				<0.001	0.997
Yes	98(80.3)	139(80.3)	237(80.3)		
No	24(19.7)	34(19.7)	58(19.7)		
**Oral hypoglycemic**				2.036	0.154
Yes	31(25.4)	32(18.5)	63(21.4)		
No	91(74.6)	141(81.5)	232(78.6)		
**Sedatives**				0.120	0.913
Yes	6(4.9)	9(5.2)	15(5.1)		
No	116(95.1)	164(94.8)	280(94.9)		
**NSAID**				13.691	<0.001*
Yes	71(58.2)	63(36.4)	134(45.4)		
No	51(41.8)	110(63.6)	161(54.6)		
**Anticholinergic**				5.624**	0.017*
Yes	1(0.8)	11(6.4)	12(4.1)		
No	121(99.2)	162(93.6)	283(95.9)		
**Corticosteroid**				1.420**	0.513
Yes	0(0.0)	2(1.2)	2(0.6)		
No	122(100.0)	171(98.8)	293(99.4)		

* Statistically significant, **Fisher’s, non-steroidal anti-inflammatory drugs (NSAIDs)

**Table 2.1:** bivariate analysis of falls and sociodemographic/medical history factors

Characteristics	Fallers n=122(%)	Non-fallers n=173(%)	Total N=295(%)	χ^2^	P-value
**Number of drugs co-administered**				0.007	0.933
Less than three	102(83.6)	144(83.2)	246(83.4)		
Three and above	20(16.4)	29 (16.8)	49(16.6)		
**Carpets/rugs**					
Torn	22(18.0)	10(5.8)	32(10.8)	11.106	0.001*
Not torn	100(82.0)	163(94.2)	263(89.2)		
**Mats**					
Slippery	63(51.6)	19(11.0)	82(27.8)	58.925	<0.001*
Not slippery	59(48.4)	154(89.0)	213(72.2)		
**Chairs**					
Without armrest	34(27.9)	26(15.0)	60(20.3)	7.280	0.007*
With armrest	88(72.1)	147(85.0)	235(79.7)		
**Wires/cord**					
Exposed on path	39(32.0)	20(11.6)	59(20.0)	18.621	<0.001*
Not exposed	83(68.0)	153(88.4)	236(80.0)		
**Bathroom floor**					
Slippery	48(39.3)	28(16.2)	76(25.8)	20.064	<0.001*
Not slippery	74(60.7)	145(83.8)	219(74.2)		
**Foot abnormality**					
Yes	28(23.0)	26(15.1)	54(18.4)	2.922	0.087
No	94(77.0)	147(84.9)	241(81.6)		
**Shoe soles**					
Uneven soles	42(34.4)	17(9.8)	59(20.0)	27.060	<0.001*
Normal soles	80(65.6)	156(90.2)	236(80.0)		
**Shoe soles**					
Slippery	51(41.8)	23(13.3)	74(25.1)	30.942	<0.001*
Not slippery	71(58.2)	150(86.7)	221(74.9)		
**Postural hypotension**					
Yes	14(11.5)	20(11.6)	34(11.5)	0.001	0.982
No	108(88.5)	153(88.4)	261(88.5)		
**Visual acuity**					
Normal vision	56(45.9)	89(51.4)	145(49.2)	0.880	0.348
Low vision	66(54.1)	84(48.6)	150(50.8)		
**Time up and go test**					
Freely mobile	50(41.0)	99(57.2)	149(50.5)	7.550	0.006*
Impaired mobility	72(59.0)	74(42.8)	146(49.5)		
**BMI**					
Underweight	4(3.3)	7(4.0)	11(3.7)	0.266**	0.977
Normal weight	69(56.6)	95(55.0)	164(55.6)		
Overweight	31(25.4)	43(24.8)	74(25.1)		
Obese	18(14.7)	28(16.2)	46(15.6)		
**PCV**				0.805	0.370
Normal	110(90.2)	161(93.1)	271(91.9)		
Anemia	12(9.8)	12(6.9)	24(8.1)		

* Statistically significant, **Fisher’s, body mass index (BMI), packed cell volume (PCV)

After adjusting for the effects of those factors which were significantly associated with falls at the bivariate level, the predictors of falls found among the study participants were age (≥ 80 years) (AOR=4.424, CI=1.192-16.424), those with a history of dizziness (AOR=0.334, CI=0.138-0.810), use of shoes with uneven (AOR=0.337, CI=0.133-0.851) and or slippery soles (AOR=0.392, CI=0.162-0.948), having slippery mats (AOR=0.086, CI=0.039-0.192) and wires/cords (AOR=0.306, CI=0.132-0.705) exposed on the pathways as seen in Table 3.

**Table 3:** logistic regression analysis for the association between risk factors and fall

Factors	AOR	95% CI	P-value
Age (≥ 80) years	4.424	1.192-16.424	0.026*
Male sex	1.252	0.549-2.857	0.593
No education	5.860	0.210-163.908	0.298
Unmarried	0.727	0.304-1.743	0.475
Diabetes mellitus	0.848	0402-1.787	0.665
Chronic joint pain	0.568	0.264-1.222	0.148
Dizziness	0.334	0.138-0.810	0.015*
NSAID	0.499	0.247-1.008	0.053
Anticholinergic	2.864	0.262-31.273	0.388
Torn carpets/rugs	0.578	0.209-1.601	0.292
Slippery mats	0.086	0.039-0.192	<0.001*
Slippery bathroom floor	1.046	0.469-2.335	0.912
Wires/cords exposed on pathways	0.306	0.132-0.705	0.005*
Uneven shoe soles	0.337	0.133-0.851	0.021*
Slippery shoe soles	0.392	0.162-0.948	0.038*
Chair without armrest	0.634	0.286-1.405	0.261
Impaired mobility (TUGT)	0.729	0.350-1.519	0.399

* Statistically significance, adjusted odd ratio (AOR), confidence interval (CI), non-steroidal anti-inflammatory drugs (NSAIDs)

## Discussion

**statement of principal findings:** the prevalence of falls among the study participants was found to be 41.4%. This prevalence resembles the 48.8% reported by Tsai *et al*. [10]. The similarities could be because of similar study settings (hospital-based), study design, and method of data collection. It was higher than the 21.4% reported by Adebiyi *et al*. [8] and 24.2% reported by Abdulraheem *et al*. [5] from north-central and south-western parts of Nigeria. The differences observed may be attributed to the study setting whereby this study was conducted in hospital where the respondents were mainly patients with relatively higher prevalence of diseases and use of medications which are established risk factors of falls compared to relatively healthier community dwelling older adults studied by the other authors. A higher prevalence of up to 66.7% was reported among elderly people living in a long-term care institution, perhaps due to advanced age and frailty that characterized the elderly people living there. A much higher prevalence of 60% was reported among older adults (>90 years) in the United Kingdom. Even though, one would expect a higher prevalence because the respondents were much older than this study, the methodology (prospective component) also limited the effect of recall bias and under-reporting phenomenon that characterize retrospective studies.

The prevalence of fall injuries in this study was 25.4%. This was similar to the 22.2% reported by Adebiyi *et al*. [8] in south-western Nigeria, this could be due to similarities in sociodemographic characteristics. A higher prevalence of 50%, 71.2%, and 79.3% was reported among the elderly in long-term care institutions [11], physiotherapy clinics [12], and inpatients [10] respectively. These findings were not surprising given that more severe morbidities and frailty would be expected among this cohort of elderly vis-à-vis patients recruited from physiotherapy clinics. Interestingly, as low as 1% and 6.6% were reported in South Africa and India respectively [13]. This could be because the studies were community-based and the cut-off age of 10 years was used. This further emphasized the influence of age on fall injuries.

The pattern of fall injuries seen in this study were swellings and pain at 31.1%, bruises at 14.8, fractures at 13.1%, and lacerations at 2.5%. This pattern was similar to those reported by Adebiyi *et al*. in southwestern Nigeria [8]. This differed from those reported in a 15-year review of 87 publications from 25 countries, of which the most common injuries were fractures (37.9%) involving the hip joint, others were head injuries; traumatic brain injury, lacerations, dislocations, sprains, and hematomas [14]. These differences were mainly due to the research settings and design. Almost 40% of the falls in this study were perceived as nothing significant. The inertia to report a fall may not be unconnected to the belief that a fall is a sign of old age and is accepted as ‘’normal’’ or due to the stigma attached to it as a sign of weakness. This is problematic because a big window of opportunity for early detection, treatment, and prevention of future occurrence is lost, leading to a vicious cycle.

The commonest cause of fall injuries was tripping, followed by slipping and occurred while walking in a well-lit area according to this study. This corroborated similar findings reported by Adebiyi *et al*. and CDC and most fall injuries occurred inside or around the outside of the home [4,8]. The predictors of falls in this study were older age, history of dizziness, use of shoes with uneven and/or slippery soles, and having slippery mats and wires/cords exposed on the pathways. This study found that older adults (≥ 80 years) are more than 400% likely to experience falls compared to those 60-69 years. This could be due to physiological decline in cardiovascular, neurological, visual, and musculoskeletal systems all of which increase the risk of falls [7]. This was similarly reported by the CDC report in USA, the WHO report on Global aging and the epidemiological review of falls in older age by the Centre for Research in Geriatric Medicine [11,15,16]. In contrast, some researchers found no greater risk of falls and fall injuries across age categories [4,17]. They attributed the higher risk of falls and injuries in those 85 years and older to the deterioration of overall health status and not due to factors intrinsic to aging.

History of dizziness was also an independent predictor of falls. This supported the finding from similar studies [4,8] who found that those with a history of dizziness were 160% and 180% more likely to fall. This further demonstrated the link between age-related vestibular dysfunction; gait impairment and falls [7]. Using shoes with uneven soles and slippery soles were found to increase the risk of falls by 30% and 15% respectively. This is because these shoes have less resistance and hence predispose the wearers to falls. This finding was similarly reported in Thailand and Boston which reported that those who had injurious falls at home were more likely to be shoeless or wearing slippers compared to those who were wearing other shoes at the time of the fall [18,19]. This is not surprising as slips (41.8%) and trips (49.2%) are the most common cause of falls in this study and footwear influences balance and subsequent risk of slips, trips, and falls by altering the somatosensory feedback.

The presence of cords and wires exposed on the pathways was also an independent predictor of falls in this study. The erratic power supply and the rampant use of generators as a source of power in this study area and consequently having cords and wires exposed on the pathway may have also contributed. This corroborated Pfortmueller *et al*. who reported that patients who had one or more environmental hazards were more likely to fall in the last 3 months [20]. In contrast, some authors reported that the existence of home hazards alone is insufficient to cause falls [21]. Rather, the interaction between an older person´s physical abilities and their exposure to environmental stressors appears to be more important. However, environmental modifications have been proven to be an effective fall prevention strategy, indicating the important role they play in the causation of falls [7,22,23].

**Further research:** this should focus on research evidence for effective strategies to reduce falls. There is a clear need for more research or initiatives, particularly in the areas of surveillance using a more robust research design and methods. In addition, more research is needed, including a larger trial, to further investigate effects of psychotropic drug on falls. Furthermore, clinical trials on the use of non-slip footwear; There are many initiatives and products that entrepreneurs, businesses, or organizations could develop and promote which could lead to improved choices for the elderly and could potentially decrease the impact of falls.

**Limitations of the study:** data were self-reported (over 12 months) and therefore, subject to information bias. The study did not include persons presenting to the emergency department and those on admission who are at higher risk for falls and related injuries, this may have underreported the prevalence and may affect the generalizability of the study. Also, the exclusion of subjects who were unable to walk or those with limb amputation may have excluded individuals with physical and mental frailty who are at increased risk of falls which led to an underestimate of the prevalence of falls and related injuries in this study.

## Conclusion

This study has provided an insight on magnitude of falls and its related injuries, which is a major health concern, especially among the elderly population. The profile of high-risk elderly patients found in this research could be used to prioritize patients for screening, which is a safe and cost-effective tool in the primary care setting. Based on these, physicians should be aware of this phenomenon and be proactive in asking, assessing, and assisting these elderly patients with fall experiences during clinical encounters and provide family-oriented, evidenced-based care that will break the cycle of silence and fear, reduce the burden of fall injuries which will improve their quality of life.

### What is known about this topic

- *Falls in the elderly is of public health concern;*
- *The prevalence of falls and fall injuries among the elderly in some parts of Nigeria*.

### What this study adds

- *The prevalence of falls in the Northern part of Nigeria is high (41.4%);*
- *Some of the important determinants of falls among the elderly in northern Nigeria are related to the environment, these include having slippery mats, as well as wires/cords exposed on the pathway;*
- *The foot wears with uneven slippery soles is also an important determinant of falls in the elderly*.

## References

[1] World Health Organisation (2021). Falls.

[2] Bergen G, Stevens MR, Burns ER (2016). Falls and Fall Injuries Among Adults Aged ≥65 Years United States. MMWR Morb Mortal Wkly Rep.

[3] Catharine RG, Cyrus C, Avan AS (2016). Prevalence and risk factors for falls in older men and women: The English Longitudinal Study of Ageing. Age and Ageing.

[4] Kalula SZ, Ferreira M, Swingler GH, Badri M (2016). Risk factors for falls in older adults in a South African Urban Community. BMC Geriatr.

[5] Abdulraheem IS, Salami SK, Bawa MK, Abdulrahem KS (2013). Prevalence and Risk Factors for Fall in Older Adults in a Nigerian Urban Community. Texila International Journal of Public Health.

[6] Rubenstein LZ (2016). Falls in the elderly. Merck Manual Professional version.

[7] Ambrose AF, Paul G, Hausdorff JM (2013). Risk factors for falls among older adults: A review of the literature. Maturitas.

[8] Adebiyi AO, Uchendu OC, Ikotun OT, Oluleye OW, Olukotun OP (2009). Falls and outcomes among old people in rural dwelling. Ann Ibadan Postgrad Med.

[9] Xavier M, Baptista H, Mendes JM, Magalhães P, Caldas-de-Almeida JM (2013). Implementing the World Mental Health Survey Initiative in Portugal-rationale, design and fieldwork procedures. Int J Ment Health Syst.

[10] Tsai LY, Tsay SL, Hsieh RK (2014). Fall Injuries and Related Factors of Elderly Patients at a Medical Center in Taiwan. Int J Gerontol.

[11] Araûjo AH, Patrício AC, Ferreira MA, Rodrigues BF, Santos TD, Rodrigues TD (2017). Falls in institutionalized older adults: risks, consequences, and antecedents. Rev Bras Enferm.

[12] James K, Gouldbourne J, Morris C, Eldemire-Shearer D, Mona J Falls and fall prevention in the elderly: insights from Jamaica. Mona. Department of Community Health and Psychiatry, Mona Ageing and Wellness Centre, University of the West Indies, undated.

[13] Williams JS, Peltzer K (2015). Risk factors and disability associated with low back pain in older adults in low-and middle-income countries. Results from the WHO study on global AGEing and adult health (SAGE). PLoS One.

[14] Terroso M, Rosa N, Torres A, Simoes R (2014). Physical consequences of falls in the elderly: a literature review from 1995 to 2010. Eur Rev Aging Physical Activity.

[15] Centers for Disease Control and Prevention (2012). Current Capture: CDC - Older Adult Falls - Preventing Falls Among Older Adults - Home and Recreational Safety - Injury Center.

[16] Peel N, Revue L (2011). Epidemiology of Falls in Older Age. Can J Aging.

[17] Grundstrom AC, Guse CE, Layde PM (2012). Risk factors for falls and fall-related injuries in adults 85 years of age and older. Arch Gerontol Geriatr.

[18] Kesley JL, Procter-Gray E, Uyen-Sa D, Li W, Kiel D, Hannan M (2010). Footwear and falls in the home among older individuals in the Maintenance of Balance, Independent Living, Intellect, and Zest in the Elderly of Boston Study. Foot Sci.

[19] Kuhirunyaratn P, Prasomrak P, Jindawong B (2013). Factors related to falls among community dwelling elderly. Southeast Asian J Trop Med Public Health.

[20] Pfortmueller CA, Kunz M, Lindner G, Zisakis A, Puig S, Exadaktylos AK (2014). Fall-related emergency department admission: Fall environment and settings and related injury patterns in 6357 patients with special emphasis on the elderly. Sci World J.

[21] Waldron N, Hill AM, Barker A (2012). Falls prevention in older adults: assessment and management. Australian family physician.

[22] Salzman B (2010). Gait and Balance Disorders in Older Adults.

[23] Marks R (2014). Falls Among the Elderly: Multi-factorial Community-based Falls-Prevention Programs. J Aging Sci.

